# Labeling Nutrition-Sensitive Food Chains: A Consumer Preference Analysis of Milk Products

**DOI:** 10.3389/fnut.2020.00158

**Published:** 2020-09-15

**Authors:** Joshua Wesana, Xavier Gellynck, Manoj K. Dora, Lucy Muyama, Emma Mutenyo, Ahikiriza Elizabeth, Edmond Kagambe, Hans De Steur

**Affiliations:** ^1^Food and Markets Department, Natural Resources Institute, University of Greenwich, Kent, United Kingdom; ^2^Department of Agricultural Economics, Faculty of Biosciences Engineering, Ghent University, Ghent, Belgium; ^3^College of Business, Arts & Social Sciences, Brunel Business School, Brunel University, London, United Kingdom; ^4^School of Education, Mountains of the Moon University, Fort Portal, Uganda

**Keywords:** nutrition sensitive value chain, labeling, consumer preference, conjoint analysis, dairy sector, Uganda

## Abstract

While nutrition-sensitive value-chain approaches are strongly advocated, studies on consumer preferences for such interventions are lacking. This study aims to fill this gap by examining a nutrition-sensitive chain labeling scheme, using the Ugandan dairy sector as a case. A survey was conducted among 250 consumers, primarily eliciting perceptions of the importance of a nutrition-sensitive chain label compared to nutrition claims/facts. In addition, a choice-based conjoint experiment was designed with nutrition label, brand, fat content, and price as attributes. Findings show that nutrition-sensitive chain labeling was more positively perceived by consumers than by nutrition claims/facts. Ordered logistic regression analysis indicated that BMI, nutrition knowledge, and label use influenced consumers' perceived importance of a nutrition-sensitive chain label relative to sex, age, children, and milk purchase frequency for nutrition claims/facts. This is confirmed by the higher utilities for the nutrition-sensitive chain label in our conjoint experiment. Future research should focus on the integration of nutrition-sensitive chain labeling with existing labels in a way that promotes candid interpretation by consumers. Industrial and policy actors in the agri-food sector can use these findings to innovate and regulate appropriate labeling schemes in the context of nutrition-sensitive value chains.

## Introduction

At a time when hunger is manifested among 821 million undernourished people (1), agriculture for nutrition has increasingly become a key intervention area for the second Sustainable Development Goal (SDG 2) (2). This reflects a gradual shift from contemporary food systems, which mainly focus on increased calorie production, to nutrition-sensitive agriculture, which builds upon value-added systems that deliver a healthy diet to consumers (3, 4). With this, the number of people facing food deprivation is expected to reduce in a more sustainable manner (5).

Whereas, the impact of agriculture on nutrition is often questioned (6, 7), inclusion of a food value chain perspective can further strengthen the effectiveness of nutrition-sensitive agriculture (8). A nutrition-sensitive value chain approach is one that “explicitly incorporates nutrition objectives and indicators into agriculture and addresses the utilization dimension of food and nutrition security” (3), thereby underpinning chain activities or processes that consider the nutritional status of food as a consumer benefit (9). As such, a value-chain contribution to nutritional outcomes is possible because value-chain approaches provide opportunities to retain and add nutritional value or prevent loss along the food chain (10, 11). Thereby, well-planned and inclusive agri-food value-chain interventions have high potential to promote supply and consumption of nutritious foods to sustainably reduce hunger (9, 12–14). Nevertheless, the rhetoric with regard to a shift to nutrition-sensitive value chains remains centered around the potential that exists, and pathways through which value chains could impact nutritional outcomes are still underutilized (15). In previous studies on nutrition-sensitive agriculture, particularly staple crops enriched with micronutrients, focus has mainly been on cost-effectiveness and hardly on value-chain development (16–18). This research gap can explain the existence of underdeveloped nutrition-sensitive value chains and their limited coverage in target countries (12, 19).

Nevertheless, effective collaboration among food value-chain actors, enabled by suitable supply-based agri-food policies, can foster the development of value chains for nutrition benefits (9, 20). Concentrating solely on the supply side, however, could limit the sensitivity of food systems to nutrition outcomes. Consumers are also important actors, and successful development of nutrition-sensitive agri-food systems largely hinges on their demand for nutritious food (14, 21). This demand is an incentive for upstream-chain actors to initiate measures that promote nutrient retention or addition as well as prevention of losses in food and nutrients (14). Thereby, consumer awareness can be a prerequisite needed to trigger a transformation among value-chain actors to consider food nutritional quality at all stages of the food chain (10). In the domain of nutrition-sensitive agriculture, so far few studies have summarized evidence on consumer preference for staple foods enriched with vitamins and minerals (12, 16, 22), though no attention has been given to consumer perceptions toward nutrition-sensitive value chains *per se*, in a farm-to-fork perspective (23–25).

When it comes to consumer food perceptions, an important distinction has to be made between product- and process-oriented quality, illustrated by either intrinsic (e.g., taste, flavor, texture, nutrient content, organic) or extrinsic (e.g., label, price, brand, package) attributes (26). As with many intrinsic attributes, nutrition-related information is generally communicated using extrinsic attributes such as labeling schemes, provided the information presented is neither confusing nor difficult to interpret (27, 28). This is certainly the case for the nutritional value of food products whereby nutrient information needs to be signaled since it can neither be observed nor easily experienced by consumers before or after purchase of food (10, 29). Through provision of information on nutritional facts or claims, nutrition labeling influences consumers' food-purchase decisions (30–32) as well as their willingness to pay (22, 33–35). Therefore, nutrition labels benefit both actors and consumers, i.e., as a cost-effective marketing tool, and, in the case of nutrition-sensitive interventions, can provide considerable return to the investment accrued by value-chain actors, while promoting healthy food eating behavior among consumers (27, 36).

Furthermore, value-chain interventions that aim at increased nutritional value of food as a consumer benefit underline process-oriented quality of food production (9, 37). This is so because approaches for nutrition benefits leverage agri-food supply chains to gain nutritional value. As such, consumers need information about the nutritional state in which food is produced, stored, distributed, and retailed before a purchase or consumption decision is made. Nutritional value, once communicated as a credence attribute, builds trust among consumers to confidently ascertain whether nutrition objectives were holistically considered along the entire value chain (3, 38, 39). This reasoning is similar to other approaches (e.g., fair trade, animal welfare, free range or organic), which also illustrates the process of food production (37, 40, 41). Therefore, in the context of nutrition-sensitive value chains it is also innovative to apply process-oriented communication toward consumers (8, 42).

This study aims to evaluate consumer preferences for a nutrition-sensitive chain-labeling scheme by (1) investigating the perceived importance compared to existing product-oriented nutrition facts or claims, (2) identifying differences in what determines the perceived importance, and (3) analyzing the trade-offs made for these nutrition labels in the presence of other product attributes. The focus on consumer preferences follows recent recommendations for research on nutrition-sensitive agriculture to first be initiated at the consumption rather than supply level, in order to better understand the factors that influence choices for healthy foods (11, 43). To our knowledge, this is the first study to investigate preferences as far as signaling nutrition-sensitive food production and supply is concerned (8, 9). It specifically underpins the special issue topic on “Sustainable Development Goals (SDGs): Impact on Nutrition” by promoting the use of novel consumer-oriented product communication strategies to improve the demand for nutrient-rich foods and healthy diets that are produced and supplied through value chains that aim to secure nutritional value as a consumer benefit throughout the chain. Hence, we make specific contributions to nutrition-sensitive value-chain literature, by recognizing that existing labeling schemes signal nutritional value added after the processing stage of the chain and so not applicable to nutrition-sensitive value chains, which encompass value added or retained at all stages of the chain (9). As such, this study proposes a way by which such an agri-food chain-based nutritional attribute that is neither explicitly observed nor easily experienced by consumers can be communicated to simulate demand for nutrient-rich foods. There is neither a process-oriented labeling scheme targeting nutritional value that spans the entire food chain (37) nor studies that assess perceptions among consumers with regard to nutrition information of value-chain processes (11, 44), unlike existing nutrition information on food products (27, 45). Therefore, this study illustrates and underpins a strategic conception of a labeling scheme, tailored to agri-food value chains for nutrition benefits, and uses dairy products in Uganda as a case. The dairy sector was selected mainly because milk is an important source of essential nutrients needed for improved nutrition and hence fits within the concept of nutrition-sensitive value chains. In 2011, the annual per capita consumption of milk in Uganda was approximated at 35 liters, but it has since increased to 54 liters (46, 47). This can be attributed to a number of interventions that have been implemented by government and other organizations to increase milk production in the country. Milk is produced by either local breeds or Holstein Friesian crossbreed cows and is mainly supplied through informal and formal market channels. While the informal channel is the largest, it is not well-organized and milk is sold directly to consumers with no form of processing done. However, the formal channel is more structured and is made up of milk processors, wholesalers, and retailers (47). As a consequence, the formal supply chain allows each actor to obtain an economic value that is generally higher than in the informal channel. There are over 40 milk-processing plants, producing a variety of products such as UHT milk, yogurt, pasteurized milk, powdered milk, cheese, butter, ice cream, and ghee (48). Their milk products are largely consumed locally, but a proportion is exported to neighboring countries, even though the local demand for milk is higher than the available supply.

## Methodology

### Survey

A survey was conducted among 250 adult consumers, interviewed at 10 retail outlets in Kampala-Uganda, during July to August 2016 by two interviewers. A convenient sample of respondents was used, and each was invited by an interviewer to participate in the study. They were first informed about the general purpose of the study, stating that it focuses on nutrition labels, and were required to give verbal consent for participation.

A pre-tested structured questionnaire (Supplementary Material) was used for data collection, and it comprised three sections. The first section elicited characteristics of respondents including age, sex, level of education, occupation, having children <5 years of age, weight, and height, in line with previous literature that showed their influence on perceptions and use of nutrition labels (27, 30, 49–54). The next section inquired about purchase frequency of milk products, label use, and nutrition knowledge among respondents. A reference period of 7 days was used for respondents to state the number of days they purchased milk or its products. Adopted from Hess et al. (55), label use was measured by asking respondents how often they used food labels to judge if food purchased was healthy (5-point scale; 1—never to 5—very often). In the context of this study, the term healthy was asked in way that aligns with nutritional healthiness as could be inferred by consumers while using nutrition labels (30). Subsequently, label use was transformed into frequent and infrequent users during analysis so as to reduce the number of empty cells. Based on recommendations to improve the assessment of nutrition knowledge made in a recent review by Miller and Cassady (56), an objective tool comprising 10 true/false statements about the nutrient content of food was adopted from the validated nutrition knowledge questionnaire of Dickson-Spillmann et al. (57). Statements elicited included the following: (1) a balanced diet implies eating all foods in the same amounts; (2) fat contains more calories than the same amount of protein; (3) the same amount of fat and sugar contains equally many calories; (4) brown sugar is much healthier than white sugar; (5) to eat healthily, you should eat less fat, you may not have to eat fruits, and vegetables; (6) if you have eaten high-fat foods, you cannot reverse the effects by eating fruits; (7) fat is always bad for your health; you should therefore avoid it as much as possible; (8) for a healthy nutrition, dairy products should be consumed in the same amounts as fruit and vegetables; (9) the health benefit of dairy products does not only lie in the supply of proteins; and (10) skimmed milk contains less vitamins than full-fat milk. Thereby, the numbers of correct responses were converted into summated scores, which were then categorized into low, intermediate, and high levels of nutrition knowledge. Transforming nutrition knowledge into a categorical variable did not affect the observed effects, and it also facilitated the presentation of results. This section was complemented with questions about perceived importance of (1) nutrition claims/facts and (2) the proposed nutrition-sensitive chain label for milk products. Perceived importance for both constituted the dependent variables of interest in this study and was measured on a 5-point Likert scale (1—not important, 5—very important) similar to a recent study by Cavaliere et al. (49), which was an improvement from a previous study that used a dichotomous scale (58).

The last section was based on a conjoint experiment, developed through the use of Sawtooth Software (version 7), following procedures of previous consumer studies (59–61). The purpose for carrying out the conjoint experiment was to mimic a potential purchase situation where a consumer makes trade-offs based on more than one product attribute and substantiate prior observed consumer perception of labels. Thereby, four attributes and corresponding levels were used in this study. Prior selection of these attributes was based on previous literature on consumer preference of food attributes (38, 40, 62) and further validated with Focus Group Discussions (FGDs) among consumers in Uganda. Thereby, two FGDs were conducted with five and six participants that were recruited locally. Discussions with participants were guided by open-ended questions that aimed at eliciting their experiences while buying food products, with a particular focus on milk products. Thereby, the first attribute was the type of label consisting of either nutrition claim/fact, nutrition-sensitive chain label or no label, as levels. Because a label signaling nutrition sensitivity of a value chain did not exist yet on food products, a prototype was designed for the purpose of the experiment. Its development was guided by other existing production process labels that target the food chain (37, 63), but with an additional consideration of nutrition sensitivity. Second attribute was brand comprising of two levels depicted by the top dairy product brands in the country (i.e., Fresh Dairy and Jesa). During the development of the tool, participants largely referred to these two brands while buying milk products. Furthermore, using brand as an attribute was meant to enhance the presentation of product concepts in a way that closely mimicked a real product encountered during a purchase situation. This eased data collection without placing a lot of thought demands on respondents. The next attribute was nutrient content, which had four levels based on the fat content of milk present on the market (i.e., whole, semi-skimmed, low fat, and skimmed milk). Using fat content to represent the attribute “nutrient content” was also meant to facilitate data collection since milk products in Uganda are already labeled as whole, semi-skimmed, low fat, and skimmed milk. This had no implication on the nutritional healthiness of food, and its use was not related to what the concept of nutrition sensitivity entails. Therefore, fat content in this study should not be viewed as a possible disqualifying nutrient present in food (64). Price was also included as the last attribute with three levels in reference to the current market prices of milk in Uganda (i.e., $ 0.67, $ 0.73, and $ 0.78).

**Figure 1:**
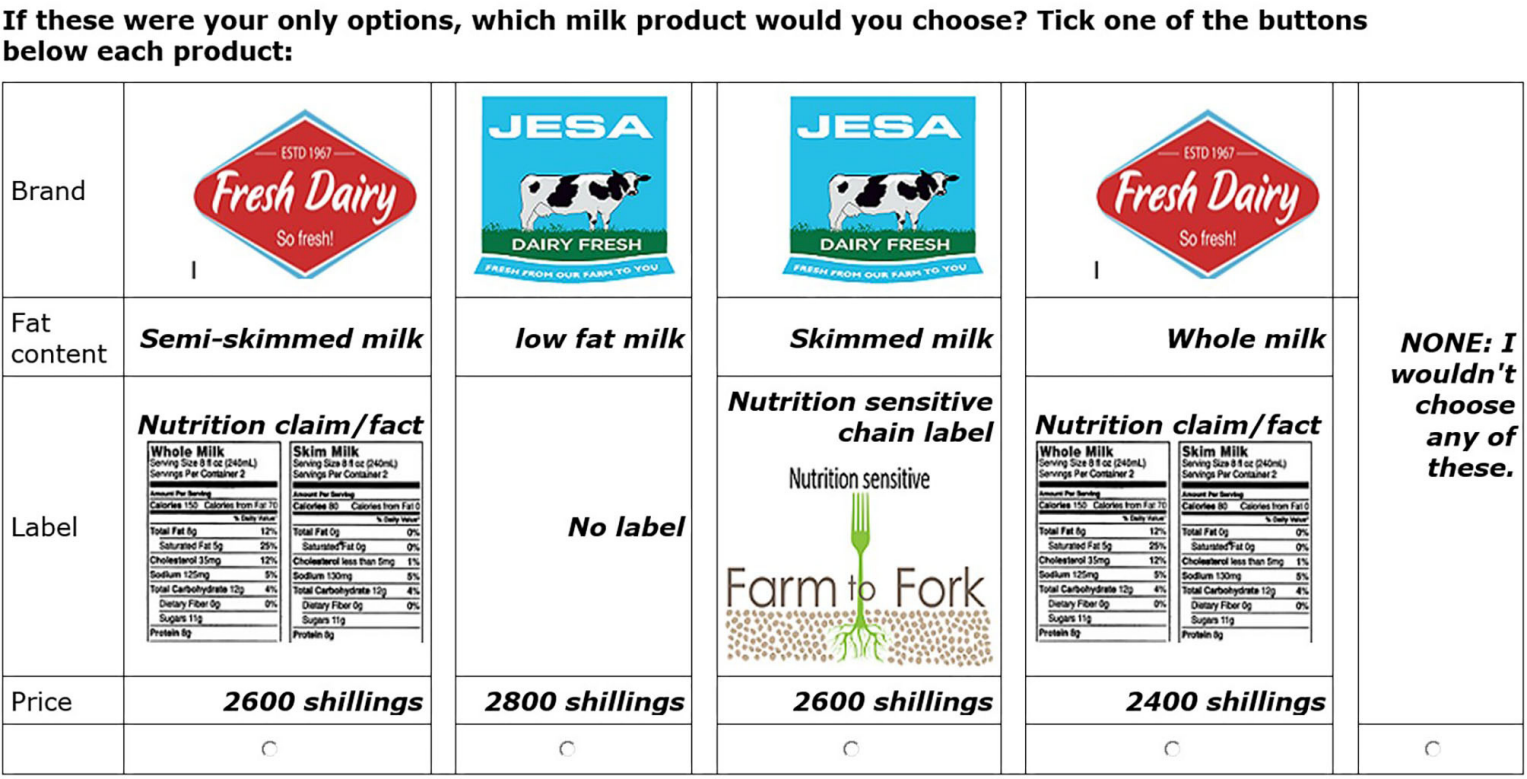
An illustration of a choice set used in the conjoint experiment.

Using a choice-based conjoint approach (65), an experimental design was developed, including eight choice sets, each with four product concepts made up of a combination of random attributes and levels. A none option was included in order to give a respondent the chance to make a choice if all the four product concepts were not preferred. This was intended to simulate a real purchase situation (66–68). Figure 1 shows one of the choice sets that were used in this study. Because of the context (i.e., consumers interviewed at retail outlets), a paper-and-pencil approach was applied using face-to-face interviews.

### Analysis

Data was analyzed using both SPSS version 23 and Sawtooth Software version 7. Descriptive statistics were computed primarily for respondent characteristics. Chi-square tests were used to assess if respondents, categorized as frequent or infrequent users of food labels, differed across other characteristics at the 5% level of significance. Before further analysis, variables were checked for multi-collinearity using correlation analysis and variance inflation factors (VIF). No independent variable was found to be collinear to another, and all were used in subsequent regression analysis. For multivariate analysis, two models were executed to investigate the effect of respondent characteristics on perceived importance of nutrition claims/facts and the proposed nutrition-sensitive chain labels, using ordered logistic regression. All respondent characteristics were included in the models, and obtained coefficients were reported in form of odds ratios to facilitate interpretation. The suitability of ordered logistic regression was assessed based on the test of parallel lines, from which the non-significance of *p*-values indicated that data satisfied the assumption of proportional odds (i.e., for facts/claims; *p* = 0.453, nutrition-sensitive chain; *p* = 0.059) (69). Because data was collected from retail outlets, differences between results obtained using normal and cluster robust standard errors were checked. As such, results were consistent, hence estimates reported as well as conclusions made in this study are based on normal standard errors. Further, as part of conjoint analysis, counts and utilities for each level of an attribute were extracted using the Sawtooth Software Market Research Tools (SMRT). Additional analysis involved computation of overall attribute “importances” in line with previous studies (59, 61). Counts were represented as proportions and stood for the number of times; for each attribute level, a product concept was chosen out of the total number of times that level appeared in all choice sets. On the contrary, utilities indicated preferences between levels of the same attribute; “importances” illustrated the relative difference or contribution each attribute made to the total utility of a product. A segmentation of conjoint utilities and counts with regard to respondent characteristics was done to compare with results obtained from the regression analysis.

## Results

### Characteristics of Respondents

Findings in Table 1 show that female respondents were on average more represented than male respondents, which corresponds with the notion that females normally make household food purchases in this context. Majority (56%) of respondents were below 30 years of age while there were relatively few (i.e., 11.6%) that were over 40 years of age. This age distribution can explain why approximately a similar proportion (55.6%) reported having children aged 5 years or below. Furthermore, there were almost 70% of respondents with an advanced education at a university level, aligning with three in four respondents that were either employed in public or private sector.

**Table 1:** Characteristics of respondents classified by food label use.

**Variable**	**Full sample *n* (%)**	**Label use**, ***n*** **(%)**		***p*-value**
		**Frequent users *n* = 161**	**Infrequent users *n* = 89**	
Sex				
Male	107 (42.8)	67 (41.6)	40 (44.9)	0.611
Female	143 (57.2)	94 (58.4)	49 (55.1)	
Age				
29 and below	140 (56.0)	89 (55.3)	51 (57.3)	0.629
30 to 39	81 (32.4)	51 (31.7)	30 (33.7)	
40 and above	29 (11.6)	21 (13.0)	8 (9.0)	
Education				
Non-advanced	78 (31.2)	45 (28.0)	33 (37.1)	0.136
Advanced	172 (68.8)	116 (72.0)	56 (62.9)	
Occupation				
Not employed	57 (22.8)	37 (23.0)	20 (22.5)	0.927
Employed	193 (77.2)	124 (77.0)	69 (77.5)	
Children <5 years				
No	111 (44.4)	69 (42.9)	42 (47.2)	0.509
Yes	139 (55.6)	92 (57.1)	47 (52.8)	
BMI				
Underweight	3 (1.2)	3 (1.9)	0	
Normal	120 (50.4)	77 (48.7)	38 (43.7)	0.264
Overweight & obese	127 (46.4)	78 (49.4)	49 (56.3)	
Nutrition knowledge				
Low	19 (7.6)	11 (6.8)	8 (9.0)	0.714
Intermediate	148 (59.2)	98 (60.9)	50 (56.2)	
High	83 (33.2)	52 (32.3)	31 (34.8)	
Milk purchase Freq				
Irregular buyer	107 (42.8)	65 (40.4)	42 (47.2)	0.297
Regular buyer	143 (57.2)	96 (59.6)	47 (52.8)	

Concerning nutrition status of respondents, majority (50.4%) had a BMI categorized as normal weight, but the proportion of those who were overweight/obese was relatively similar (i.e., 46.4%), a clear indication of the current nutrition transition in developing regions. A large majority of respondents (i.e., 92.4%) exhibited intermediate and high levels of nutrition knowledge, although the former were more prominent. These respondents could give between 5 or more correct answers out of the 10 knowledge assessment items used in this study. In terms of consumer behavior related to frequency of milk purchase, the proportion of regular buyers was higher (57.2%) than that of irregular buyers (42.3%).

Two consumer segments based on label use were also identified (i.e., frequent and infrequent users of food labels). The former were relatively more than the latter (161 against 89), and similar distributions were observed across categories of gender, age, education, occupation, children, BMI, nutrition knowledge, and milk purchase frequency. However, the proportion of both frequent and infrequent users of food labels did not significantly differ across these variables (*p* > 0.05).

### Perceived Importance of Nutrition Labeling

**Figure 2:**
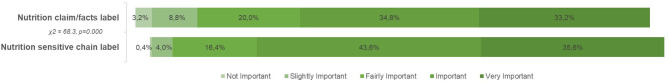
Comparison of the perceived importance of existing nutrition labels and a proposed chain-based label on dairy products.

As for the importance of food labels (Figure 2), majority of consumers considered a nutrition-sensitive chain label on food products to be more important than contemporary nutrition claims/facts (χ^2^ = 68.3, *p* = 0.000). This was true among 35.6 and 43.6% of respondents that rated the chain label as “very important” and “important” compared to the 33.2 and 34.8% for claims/facts, respectively. In addition, a higher proportion (3.2%) of respondents found claims/facts unimportant in comparison to the 0.4% for the chain label. Nonetheless, there was an overall tendency among consumers to rate both types of labels on the higher side of the importance scale.

### Determinants for Perceived Importance of Nutrition Labeling

Results of the ordered logistic regression show that four factors influence the perceived importance attached to nutrition facts/claims (Table 2). Female respondents were close to two times more likely to rate perceived importance of nutrition claims/facts at a higher level of the scale than their male counterparts. Respondents in the middle-age category (i.e., 30–39) were less likely to rate perceived importance of nutrition claims/facts at a higher scale level than those aged 40 years and above. When compared to respondents without children <5 years old, those with these children exhibited an increased likelihood to rate nutrition claims/facts as more important. Results also indicate that the more often respondents purchased milk products in a week, the higher was their perceived importance of nutrition claims/facts during a purchase situation. Other five factors (i.e., education, occupation, BMI, nutrition knowledge, and label use) did not significantly influence perceived importance of nutrition claims/facts among respondents.

**Table 2:** Determinants of perceived importance of existing nutrition labels and the proposed chain-based label on dairy products.

	**Nutrition facts & claims**			**Nutrition-sensitive chain label**		
	**OR**	**95% CI**	***p*-value**	**OR**	**95% CI**	***p*-value**
Sex						
Male (ref)	1			1		
Female	1.75	1.08–2.83	0.024^**^	1.37	0.83–2.26	0.219
Age						
40 and above (ref)	1			1		
29 and below	0.52	0.23–1.20	0.125	0.85	0.38–1.92	0.694
30 to 39	0.42	0.18–0.97	0.043^**^	0.64	0.28–1.46	0.288
Education						
Non-advanced (ref)	1			1		
Advanced	1.35	0.80–2.27	0.258	1.09	0.64–1.83	0.761
Occupation						
Not employed (ref)	1			1		
Employed	1.02	0.56–1.84	0.957	1.26	0.68–2.31	0.464
Children <5 years						
No (ref)	1			1		
Yes	1.85	1.10–3.13	0.021^**^	1.09	0.64–1.85	0.759
BMI						
Normal (ref)	1					
Underweight	2.57	0.32–20.41	0.373	0.21	0.02–2.62	0.226
Overweight & obese	1.14	0.70–1.85	0.594	1.64	1.00–2.69	0.049^**^
Nutrition knowledge						
High (ref)	1			1		
Intermediate	0.88	0.54–1.44	0.604	0.54	0.33–0.89	0.016^**^
Low	2.60	0.49–13.75	0.262	1.83	0.29–11.64	0.523
Milk purchase frequency						
Irregular buyer (ref)	1			1		
Regular buyer	2.15	1.28–3.61	0.004^**^	1.20	0.70–2.04	0.506
Label use						
Infrequent user (ref)	1			1		
Frequent user	1.32	0.82–2.14	0.254	2.24	1.36–3.70	0.002^**^

** *Significant at p < 0.05*.

*OR means odds ratio*.

*CI means confidence interval*.

Regarding the model on the importance of nutrition-sensitive chain label, three factors were significant. Overweight or obese respondents were more likely to perceive this label as important compared to normal-weight respondents. The effect of nutrition knowledge was exhibited in this model, showing that respondents with an intermediate level of nutrition knowledge had a lower likelihood to perceive the chain-based label as important in contrast to respondents with higher nutrition knowledge. Furthermore, respondents that were profiled as frequent users of food labels were over two times more likely to perceive nutrition-sensitive chain label as an important attribute than infrequent users. Surprisingly, sex, age, education, occupation, children <5 years of age, and milk purchase frequency were not significant determinants of perceived importance for this label.

### Trade-Offs for Nutrition Labels in Presence of Other Product Attributes

Findings from the conjoint experiment in Table 3 further indicate consumer preferences based on trade-offs made among product attributes during purchase. Taken as a whole, nutrition labeling as an attribute was considered the most important (71.1%), much higher than brand, fat content, and price. Furthermore, nutrition-sensitive chain label as a level to the attribute nutrition label was selected 52% of the times it appeared, higher than the other two levels of that attribute. It also contributed highest and positively to the total utility.

**Table 3:** Consumer preferences for product attributes. Attribute importance, counts, and part-worth utilities from conjoint analysis.

	**Attributes (importance, in %) & levels (counts, in % & utilities*****)***											
	**Nutrition label (71.1%)**			**Brand (11.1%)**		**Fat content (13.0%)**				**Price (4.8%)**		
	**Claim/fact**	**Sensitive chain**	**None**	**Jesa**	**Fresh Dairy**	**WM**	**SSM**	**LFM**	**SM**	**$ 0.67**	**$ 0.73**	**$ 0.78**
All respondents												
	39.1, **0.50**	52.2, **0.71**	8.7, **−1.21**	41.6, **−0.15**	58.4, **0.15**	28.1, **−0.01**	26.2, **0.13**	19.4, **−0.22**	26.2, **0.01**	33.3, **−0.08**	33.3, **0.03**	33.4, **0.05**
Sex												
Male	35.6, **0.43**	56.1, **0.80**	8.2, **−1.24**	38.7, **−0.20**	61.3, **0.20**	26.8, **−0.04**	26.7, **0.17**	19.5, **−0.22**	27.0, **0.10**	32.4, **−0.09**	33.3, **0.05**	34.2, **0.04**
Female	41.6, **0.56**	49.3, **0.64**	9.1, **−1.20**	43.7, **−0.11**	56.3, **0.11**	29.1, **0.01**	25.9, **0.10**	19.4, **−0.21**	25.6, **0.10**	34.0, **−0.05**	33.2, **0.02**	32.7, **0.05**
Age												
29 and below	41.2, **0.53**	49.6, **0.62**	9.2, **−1.16**	42.2, **−0.13**	57.8, **0.13**	29.3, **0.05**	24.4, **0.01**	19.8, **−0.21**	26.4, **0.15**	34.0, **−0.06**	33.5, **0.05**	32.5, **0.01**
30 to 39	29.0, **0.23**	61.0, **0.89**	10.0, **−1.12**	39.6, **−0.21**	60.4, **0.21**	28.7, **−0.01**	27.7, **0.24**	18.7, **−0.25**	24.9, **0.02**	33.7, **−0.08**	32.5, **−0.03**	33.8, **0.11**
40 and above	39.2, **0.56**	53.4, **0.81**	7.4, **−1.37**	43.9, **−0.08**	56.1, **0.08**	21.0, **−0.25**	30.6, **0.37**	19.6, **−0.17**	28.8, **0.04**	29.5, **−0.15**	34.1, **0.05**	36.3, **0.10**
Education												
Non-advanced	39.5, **0.52**	51.1, **0.65**	9.4, **−1.18**	45.3, **−0.07**	54.7, **0.07**	26.2, **−0.07**	27.9, **0.20**	17.1, **−0.34**	28.8, **0.21**	31.4, **−0.13**	34.3, **0.07**	34.3, **0.06**
Advanced	38.9, **0.50**	52.7, **0.74**	8.4, **−1.23**	39.9, **−0.18**	60.1, **0.18**	29.0, **0.02**	25.5, **0.10**	20.5, **−0.16**	25.0, **0.05**	34.2, **−0.05**	32.8, **0.01**	33.0, **0.04**
Occupation												
Not employed	38.2, **0.37**	49.3, **0.54**	12.5, **−0.91**	43.8, **−0.10**	56.2, **0.10**	30.8, **0.13**	25.1, **0.06**	15.7, **−0.45**	28.4, **0.26**	33.9, **−0.07**	32.6, **0.02**	33.6, **0.05**
Employed	39.3, **0.56**	53.1, **0.77**	7.6, **−1.33**	40.9, **−0.16**	59.1, **0.16**	27.4, **−0.05**	26.5, **0.15**	20.5, **−0.15**	25.6, **0.05**	33.2, **−0.08**	33.5, **0.03**	33.3, **0.05**
Children <5 years												
No	35.8, **0.36**	54.5, **0.74**	9.7, **−1.10**	43.3, **−0.09**	56.6, **0.09**	27.9, **0.01**	26.2, **0.11**	18.2, **−0.28**	27.7, **0.17**	33.1, **−0.05**	33.4, **0.05**	33.5, **0.00**
Yes	41.7, **0.62**	50.4, **0.69**	8.0, **−1.31**	40.1, **−0.19**	59.9, **0.19**	28.3, **−0.03**	26.2, **0.15**	20.4, **−0.16**	25.0, **0.04**	33.5, **−0.09**	33.2, **0.02**	33.3, **0.07**
BMI												
Underweight	25.4, **−0.21**	55.9, **0.43**	18.6, **−0.22**	30.0, **−0.29**	70.0, **0.29**	49.4, **0.95**	28.8, **0.54**	17.3, **−0.10**	4.5, **−1.39**	34.8, **−0.16**	39.1, **0.02**	26.1, **0.14**
Normal	40.4, **0.51**	49.8, **0.62**	9.7, **−1.13**	40.9, **−0.17**	59.1, **0.17**	28.5, **0.02**	26.0, **0.10**	19.5, **−0.23**	26.0, **−0.11**	33.3, **−0.09**	34.7, **0.08**	32.1, **0.01**
Overweight & obese	37.9, **0.53**	54.7, **0.81**	7.5, **−1.34**	42.5, **−0.11**	57.5, **0.11**	27.3, **−0.06**	26.4, **0.16**	19.4, **−0.20**	26.9, **0.10**	33.4, **−0.07**	31.7, **−0.02**	34.9, **0.09**
Nutrition knowledge												
Low	34.6, **0.76**	63.2, **1.38**	2.2, **−2.14**	39.7, **−0.12**	60.3, **0.12**	33.4, **0.14**	26.1, **0.28**	18.9, **−0.23**	21.6, **−0.19**	30.6, **−0.23**	30,8, **0.08**	38.6, **0.15**
Intermediate	39.3, **0.46**	50.6, **0.64**	10.1, **−1.10**	42.4, **−0.13**	57.6, **0.13**	27.9, **−0.03**	27.7, **0.20**	18.0, **−0.32**	26.4, **0.15**	33.4, **−0.08**	34.3, **0.08**	32.4, **0.00**
High	39.6, **0.57**	52.6, **0.76**	7.9, **−1.33**	40.4, **−0.18**	59.6, **0.18**	27.3, **−0.01**	23.7, **−0.04**	22.2, **−0.02**	26.8, **0.07**	33.8, **−0.06**	32.1, **0.05**	34.1, **0.11**
Milk purchase frequency												
Irregular buyer	37.6, **0.36**	51.5, **0.65**	11.0, **−1.01**	44.3, **−0.07**	55.7, **0.07**	28.9, **0.03**	26.3, **0.12**	18.3, **−0.29**	26.6, **0.14**	33.6, **−0.04**	33.9, **0.06**	32.6, **−0.02**
Regular buyer	40.2, **0.62**	52.8, **0.78**	7.1, **−1.41**	39.5, **−0.20**	60.5, **0.20**	27.6, **−0.04**	26.2, **0.14**	20.3, **−0.17**	25.9, **0.06**	33.2, **−0.11**	32.9, **0.01**	34.0, **0.10**
Label use												
Infrequent user	42.5, **0.60**	48.6, **0.59**	8.9, **−1.18**	44.8, **−0.09**	55.2, **0.09**	28.8, **0.08**	27.8, **0.19**	16.0, **−0.39**	27.4, **0.11**	32.0, **−0.12**	36.2, **0.11**	31.8, **0.01**
Frequent user	32.9, **0.35**	58.7, **0.91**	8.3, **−1.26**	39.8, **−0.19**	60.2, **0.19**	27.7, **−0.05**	25.4, **0.09**	21.3, **−0.14**	25.6, **0.10**	34.1, **−0.04**	31.6, **−0.03**	34.3, **0.07**

*WM, whole milk; SSM, semi-skimmed milk; LFM, low-fat milk; SM, skimmed milk*.

*Utilities are shown in bold*.

With respect to conjoint results stratified by respondent characteristics, female regular consumers of milk, with children below 5 years of age, selected products with nutrition claims/facts at higher proportions, and this attribute contributed positively to the total utility. This aligns with the tendency observed among consumers with this profile to consider nutrition claims/facts more important than their counterparts. As expected, consumers in the middle-age category (i.e., 30–39 years) were less likely to select products labeled with nutrition claims/facts, hence having the lowest influence on utility. This fully corresponds with the reduced likelihood to perceive nutrition claims/facts as important observed among middle-aged consumers.

Furthermore, a higher proportion of products labeled as nutrition-sensitive were selected by overweight or obese and frequent label users. This category of respondents also exhibited greater utility attributed to the presence of the nutrition-sensitive chain label compared to normal-weight and infrequent label users. These consumer characteristics also emerged as significant determinants of perceived importance of a label signaling nutrition sensitivity of food chains. Consumers with intermediate nutrition knowledge least preferred products labeled as nutrition sensitive, and this attribute marginally influenced total utility. These consumers also did not consider nutrition-sensitive labeling useful relative to more knowledgeable consumers.

With regard to other attributes used in the conjoint analysis, results from all respondents indicate that consumers preferred the Jesa brand to the Fresh Dairy brand and the former adds positively to total utility. Products with levels for both fat content (i.e., whole milk, semi-skimmed milk, and skimmed milk) and price (i.e., $0.67, $0.73, $0.78) had approximately equal proportions of selection whenever they appeared in a choice set. Nevertheless, results show that only whole milk, low-fat milk, and products costing $0.67 reduced total utility. In general, the distribution of conjoint results observed for brand, fat content, and price was the same for different categories of respondents, profiled by consumer characteristics.

## Discussion

This study explored preference among consumers toward nutrition-sensitive chain labeling, while making a reference to consumer use, and perceived importance of existing nutrition labels. Findings pointed to a high proportion of consumers that reported using nutrition labels on a regular basis. While a large body of literature about nutrition labels originates from developed countries, observations for the increased frequency of label use from a few studies conducted in low-income settings are similar to findings in our study (45). With regard to potential determinants of label use, only the effects of gender, age, and education are consistent with previous findings, while those of BMI and nutrition knowledge are contradictory (32, 70). The insignificance of these consumer characteristics on label use is surprising since previous studies have shown that nutrition label use is largely increased by having nutrition knowledge, higher education, older age, employment, and being a woman (56, 71–73). It could be interesting to further explore if motivational factors are more relevant than demographic differences to explain high label use observed in this context (74).

Regarding the importance of nutrition labeling, there was an observed inclination toward the proposed nutrition-sensitive chain label over existing nutrition claims/facts. The positive perception for the former may have been influenced by distrust among consumers toward nutrition claims (45, 75, 76). Thereby, having a label that promotes nutrition and spans the entire food chain, in a positive way, could have given consumers an impression that this strategy would be regulated by a reputable institution, rather than controlled by food industrial players only, who are considered less credible sources of information (77).

Findings further point to differences in determinants of consumer preference for the two label categories. Consumers preferring nutrition claims/facts were most likely female, aged 40 or above, with children <5 years old and regular purchasers of milk products. Such consumers who are often responsible for the well-being of their households tend to be health conscious and hence expected to make healthy food choices (49, 50). This is further emphasized in a study highlighting that female consumers who usually purchase food for other family members tend to use nutrition information regularly (74). There are also indications that those who exhibit a positive attitude toward healthy foods are more likely to pay a premium for food with a nutrition label, even in the presence of cheaper alternatives, in support of previous studies (33, 78). For the nutrition-sensitive chain label, there was an increased interest from respondents that were overweight or obese. Without previous studies looking at such a label, this finding could initially be explained in relation to other respondent characteristics observed in this study. Thereby, high levels of education and nutrition knowledge observed among these respondents might have made it easier for them to understand the concept of a nutrition-sensitive value chain and associated benefits. This is further supported by previous evidence showing that elevated BMI is associated with interest in nutrition labels (79). Results also indicated that regular users of food labels for health purposes considered nutrition-sensitive chain labels important. This complements positive attitudes among such consumers observed in previous studies, specifically dealing with similar process-oriented labels for healthy foods (40). Our findings interestingly suggest that consumers with moderate nutrition knowledge attach lower importance to nutrition-sensitive chain labels than those with higher nutrition knowledge. According to Walters and Long (80), having a high level of nutrition knowledge plays a very crucial role to facilitate information processing on food labels. This could imply that advocating for adoption of this labeling scheme might need additional targeted strategies to enhance nutrition knowledge among consumers, as recently suggested by Viola et al. (81). A starting point would be to tap into the already numerous interventions or programs that target enhancement of nutrition knowledge and seek for complementary efforts to promote nutrition-sensitive agriculture (82–84).

As it has been difficult for regulatory bodies, the food industry, and other stakeholders to find a consensus for harmonized labeling schemes with easily understandable information (85), there could be a growing threat to nutrition labeling among consumers (27). With the emergence of various labeling schemes, one would wonder if more nutrition information worsens information overload for consumers (51, 52). Current evidence, however, has illustrated increased consumer interest in having more nutrition information added to labels provided the content, format, size, and position facilitate interpretation (86, 87). This is positive, and because nutrition-sensitive chain labeling inherently combines the benefits offered by existing nutrition labels, its use as a novel approach is reasonable. In fact, our findings also suggest that existing labels are, to a given extent, still popular among consumers, even in a developing country context, in support of previous research findings (45, 88, 89). Hence, food regulators should be cautious of issues related to consumer understanding of food labels and ensure control over design and presentation of labels. A collective and evidence-based solution is needed for this threat not to linger at the expense of the consumer while stakeholders should pick interest in exploring innovative ways that appropriately combine different schemes. In the meantime, deliberate efforts that target innovative educational interventions to enhance consumer understanding of nutrition information provided on food products can be supported continuously and also backed up by pragmatic research for their effectiveness.

This study is unique, having assessed consumer preference for food labeling in the context of nutrition-sensitive chain production and supply, hence filling an existing knowledge gap on ways through which demand for nutritional added value of food chains could be promoted (8, 9). This is timely and not only of great value as a necessity to reinforce effectiveness of pathways to improve access to nutritious food but also makes a strategic contribution to the success of SDG 2 targeting food and nutrition security (8, 42). Another strength was executing a choice-based conjoint experiment, focusing on nutrition labeling in a developing country context. Such evidence is also missing but extremely needed to underline the interaction between nutrition labels and other product attributes in developing countries (9). Regardless of the study's contribution, there are some limitations worth mentioning. Because a nutrition-sensitive chain label does not exist yet on food products, there could be a possibility that results were influenced by a hypothetical bias. Nevertheless, we believe that comparisons made with existing nutrition claims/facts, using brands of milk products already on the market and also using a graphical prototype of the label helped to reduce the magnitude of this bias. Another possible weakness of this study was the urban location where the study was conducted, which limits generalization of reported results to rural contexts. However, targeting urban respondents was justified because they are the key market for pre-packaged foods, which are normally labeled, hence fitting in the study scope. It could be interesting if innovative labeling schemes are developed and adapted to rural agri-food settings. Thereby, future studies would be able to explore possible differences in preferences among consumers from both urban and rural contexts. In addition, information given to respondents before the questionnaire was administered might have introduced self-selection bias. It is likely that respondents who participated might have had prior experiences with nutrition labels. This could also have led to an effect of social desirability on study results whereby such respondents would most likely be inclined to give positive answers. Other factors such as time constraints and convenience might have affected participation especially for potential respondents that turned down the invitation to be interviewed. However, follow-up on the characteristics of non-responders was not possible and the likely impact on findings could not be determined.

## Conclusion

To establish an engagement with consumers regarding nutrition-sensitive value chains, this study explored consumer preference for a label that signals food production processes, considering nutrition objectives along agri-food value chains. Findings indicated that the proposed nutrition-sensitive chain label was considered more important than existing nutrition labels and BMI, nutrition knowledge, and label use are identified as key factors influencing this consumer behavior. These observations were also justified by results obtained from the choice-based conjoint experiment. While promoting the advent of a labeling scheme to signal value from nutrition-sensitive value chains, a consideration of its practical feasibility and success is important. Developers need evidence of not only the willingness to adopt among value-chain actors but also mechanisms to establish effective regulation and enforcement. In addition, food producers can be motivated to expand market share by leveraging observed significant consumer characteristics (e.g., nutrition knowledge and nutrition status) as target market segments of food labeled as nutrition sensitive. Furthermore, the type(s) of food chains suitable for such a label needs to be considered. Although this study used the dairy sector as a case, any food that is pre-packed could be targeted but emphasis should be put on nutrient-dense foods prone to losses, as a pathway to improve nutrition. Thereby, development of a chain-based label to signal nutrition sensitivity is a process that requires various multi-stakeholder engagements, feasibility studies, and policy commitment, as prerequisites for success. Future research should use this study as a reference for application to other food products and contexts as well as input for developing a chain-based nutrition-sensitive label. Nonetheless, this study gives relevant evidence of an enduring consumer need for information that stimulates healthy food choices, especially in the context of nutrition-sensitive food production and supply. Hence, it makes a timely contribution to limited research about nutrition-sensitive value chains in a consumer perspective.

## Data Availability Statement

The datasets generated for this study are available on request to the corresponding author.

## Ethics Statement

Ethical review and approval was not required for the study on human participants in accordance with local legislation and the requirements of the Mountains of the Moon University, Uganda. The patients/participants provided their written informed consent to participate in this study.

## Author Contributions

JW contributed to the conceptualization, development of study tools, data collection, data processing, data analysis, drafting, and revision of the manuscript. XG and MD contributed to the conceptualization, development of study tools, and revision of the manuscript. LM and EM contributed to the development of study tools, data collection, data processing, data analysis, and revision of the manuscript. AE contributed in data collection, data processing, data analysis, and revision of the manuscript. EK contributed to the development of study tools, data collection, and revision of the manuscript. HD contributed to the conceptualization, development of study tools, data analysis, and revision of the manuscript. All authors contributed to the article and approved the submitted version.

## Conflict of Interest

The authors declare that the research was conducted in the absence of any commercial or financial relationships that could be construed as a potential conflict of interest.
