# Supplementary material for: Labeling Nutrition-Sensitive Food Chains: A Consumer Preference Analysis of Milk Products
Source: Front Nutr. 2020 Sep 15;7:158. doi: 10.3389/fnut.2020.00158 (PMC7522577; doi:10.3389/fnut.2020.00158)
Supplement: Supplementary file 1 [file Data_Sheet_1.PDF]

Questionnaire Theme:

Consumer preference for a nutrition sensitive chain label

Dear,

I would like to invite you to give us your opinions for an ongoing research to get a deeper understanding how consumers react to labels on found on food products, with a special focus on nutrition labels. It will take about **20 minutes** of your precious time to answer the questions. Your personal answers will be confidentially treated and will not be shared by anyone external to the research team.

If you agree to participate in this survey, kindly provide honest and reliable information to each question.

Thank you.

Would you like to participate in this survey?

Identification number: UG_______

Location: ___________________

PART A: CHARACTERISTICS OF RESPONDENT

1.	Age of respondent	*…………………..years*	
2.	Sex of respondent	*1= Male*<br>*2= Female*	
3.	What is the highest level of education you have attained *(also specify class)*	*1= No Education*<br>*2= Primary*<br>*3= Secondary*<br>*4= University*	……………..
4.	What is your current occupation status	*1= Not employed*<br>*2= Self-employed*<br>*3= Employed by gov't*<br>*4= Employed in Private sector*	
5.	Average monthly income of respondent	*……………………………shillings*	
6.	Do you have children five years or below?	*1= Yes*<br>*2= No*	
7.	Measure and indicate respondent weight	*…………….in kg*	
8.	Measure and indicate respondent height	*…………….in cm*	

PART B: FREQUENCY OF PURCHASE, NUTRITION KNOWLEDGE, USE, SEARCH AND UNDERSTANDING OF NUTRITION INFORMATION ON MILK PRODUCTS

7. How many times a week (in 7 days) do you purchase milk products?

**Frequency/**days

8. Please indicate if the following statements are true or false

State true or false	**True/False**
A balanced diet implies eating all foods in the same amounts	
Fat *(e.g. in blue band)* contains more calories than the same amount of protein *(e.g. in beans)*	
The same amount of fat and sugar contains equally many calories	
Brown sugar is much healthier than white sugar	
To eat healthily, you should eat less fat. You may not have to eat fruits & vegetables	
If you have eaten high-fat foods, you cannot reverse the effects by eating fruits	
Fat is always bad for your health; you should therefore avoid it as much as possible	
For a healthy nutrition, dairy products should be consumed in the same amounts as fruit and vegetables	
The health benefit of dairy products does not only lie in the supply of proteins	
Skimmed milk contains less vitamins than full-fat milk	

9. Please indicate how often you use food labels during the following purchase situations.

Circle what applies	**Never (1)**	**Rarely (2)**	**Sometimes (3)**	**Often (4)**	**Very often (5)**
Judging if the food is healthy	1	2	3	4	5

10. How important do you consider having the following types of nutrition labels on milk products you purchase?

Circle what applies	**Not at all important (1)**	**Slightly important (2)**	**Fairly important (3)**	**Important (4)**	**Very important (5)**
Nutrition claims/claims	1	2	3	4	5

Now, I am going to ask you questions regarding an agri-food value chain for nutrition benefits and new nutrition label concept (nutrition sensitive chain label) that once included on the food products you purchase, unlike the labels discussed before, shows whether nutritional enhancing efforts, that target retention and addition of nutritional value or prevention of losses, are considered throughout the whole supply chain (farmer, processor, wholesaler, retailer etc.). By so doing, consumers are able to ascertain that deliberate efforts were made by actors during production and supply to enhance or sustain the nutritional quality of food (i.e. process-oriented quality). This signals credence of the food production and supply process, to specifically illustrate nutritional attributes, that are neither explicitly observed nor easily experienced by consumers. In so doing, trust and confidence among consumers interested in nutritional benefits of food can be built to promote purchase and consumption of healthy foods.

Please note that this kind of label is still under development and has not been used before on food products. The purpose of the following questions is to assess consumer views and perceptions prior to the launch of such a label. Consumer research has shown that people often respond in one way but act differently. We therefore, ask you to respond to each of the following questions based on your true perception.

11. How important do you consider having a nutrition chain oriented label (i.e. NutSen Chain label) on milk products you purchase?

Circle what applies	**Not at all important (1)**	**Slightly important (2)**	**Fairly important (3)**	**Important (4)**	**Very important (5)**
NutSen chain label	1	2	3	4	5

PART C: CHOICE BASED CONJOINT EXPERIMENT

12.

If these were your only options, which milk product would you choose? Tick one of the buttons below each product:

Brand	Fresh Dairy	JESA	JESA	Fresh Dairy	
Fat content	***Semi-skimmed/reduced fat milk***	***low fat milk***	***Skimmed/No fat milk***	***Whole/Full cream milk***	***NONE: I wouldn't choose any of these.***
Label	***Nutrition claim/fact***	***No label***	***Nutrition sensitive chain label***	***Nutrition claim/fact***	
Price	***2600 shillings***	***2800 shillings***	***2600 shillings***	***2400 shillings***	
	○	○	○	○	○

D - 1, T - Choiceset_Random1

If these were your only options, which milk product would you choose? Tick one of the buttons below each product:

Brand	JESA	JESA	Fresh Dairy	Fresh Dairy	
Fat content	***Whole/Full cream milk***	***Semi-skimmed/reduced fat milk***	***low fat milk***	***Skimmed/No fat milk***	***NONE: I wouldn't choose any of these.***
Label	***Nutrition sensitive chain label***	***No label***	***Nutrition sensitive chain label***	***No label***	
Price	***2400 shillings***	***2800 shillings***	***2800 shillings***	***2600 shillings***	
	○	○	○	○	○

D - 1, T - Choiceset_Random2

If these were your only options, which milk product would you choose? Tick one of the buttons below each product:

Brand	Fresh Dairy	JESA	Fresh Dairy	JESA	
Fat content	***Whole/Full cream milk***	***Whole/Full cream milk***	***Skimmed/No fat milk***	***Skimmed/No fat milk***	***NONE: I wouldn't choose any of these.***
Label	***Nutrition claim/fact***	***Nutrition claim/fact***	***No label***	***Nutrition sensitive chain label***	
Price	***2400 shillings***	***2400 shillings***	***2800 shillings***	***2800 shillings***	
	○	○	○	○	○

D - , T - Choiceset_Fixed1

If these were your only options, which milk product would you choose? Tick one of the buttons below each product:

Brand	Fresh Dairy	JESA	Fresh Dairy	JESA	
Fat content	Semi-skimmed/reduced fat milk	low fat milk	Whole/Full cream milk	Skimmed/No fat milk	NONE: I wouldn't choose any of these.
Label	No label	Nutrition claim/fact	Nutrition sensitive chain label	Nutrition claim/fact	
Price	2400 shillings	2800 shillings	2800 shillings	2400 shillings	
	○	○	○	○	○

D - 1, T - Choiceset_Random3

If these were your only options, which milk product would you choose? Tick one of the buttons below each product:

Brand	Fresh Dairy	Fresh Dairy	JESA	JESA	
Fat content	low fat milk	Whole/Full cream milk	Semi-skimmed/reduced fat milk	Semi-skimmed/reduced fat milk	NONE: I wouldn't choose any of these.
Label	No label	Nutrition claim/fact	Nutrition sensitive chain label	Nutrition claim/fact	
Price	2400 shillings	2600 shillings	2600 shillings	2800 shillings	
	○	○	○	○	○

D - 1, T - Choiceset_Random4

If these were your only options, which milk product would you choose? Tick one of the buttons below each product:

Brand	JESA	Fresh Dairy	Fresh Dairy	JESA	
Fat content	Skimmed/No fat milk	Whole/Full cream milk	low fat milk	Skimmed/No fat milk	NONE: I wouldn't choose any of these.
Label	Nutrition claim/fact	Nutrition sensitive chain label	Nutrition sensitive chain label	No label	
Price	2400 shillings	2600 shillings	2600 shillings	2800 shillings	
	○	○	○	○	○

D - 1, T - Choiceset_Random5

If these were your only options, which milk product would you choose? Tick one of the buttons below each product:

Brand	Fresh Dairy	JESA	Fresh Dairy	JESA	
Fat content	Whole/Full cream milk	Whole/Full cream milk	Skimmed/No fat milk	low fat milk	NONE: I wouldn't choose any of these.
Label	Nutrition claim/fact	No label	No label	No label	
Price	2400 shillings	2600 shillings	2800 shillings	2600 shillings	
	○	○	○	○	○

D - 1, T - Choiceset_Random6

If these were your only options, which milk product would you choose? Tick one of the buttons below each product:

Brand	JESA	Fresh Dairy	JESA	Fresh Dairy	
Fat content	Semi-skimmed/reduced fat milk	Semi-skimmed/reduced fat milk	low fat milk	Skimmed/No fat milk	NONE: I wouldn't choose any of these.
Label	Nutrition sensitive chain label	Nutrition claim/fact	Nutrition claim/fact	Nutrition sensitive chain label	
Price	2400 shillings	2800 shillings	2800 shillings	2400 shillings	
	○	○	○	○	○

D - 1, T - Choiceset_Random7

Please, kindly indicate your **telephone number** for further inquiry if needed about the above topic____________________

THANK YOU FOR PARTICIPATING
